# Metabolic and microbiota response to arginine supplementation and cyclic heat stress in broiler chickens

**DOI:** 10.3389/fphys.2023.1155324

**Published:** 2023-03-31

**Authors:** Giorgio Brugaletta, Luca Laghi, Marco Zampiga, Chiara Oliveri, Valentina Indio, Raffaela Piscitelli, Stefano Pignata, Massimiliano Petracci, Alessandra De Cesare, Federico Sirri

**Affiliations:** ^1^ Department of Agricultural and Food Sciences, Alma Mater Studiorum—University of Bologna, Bologna, Italy; ^2^ Department of Physics and Astronomy, Alma Mater Studiorum—University of Bologna, Bologna, Italy; ^3^ Department of Veterinary Medical Sciences, Alma Mater Studiorum—University of Bologna, Bologna, Italy

**Keywords:** broiler chicken, arginine, heat stress, metabolism, plasma, liver, breast muscle, microbiota

## Abstract

Little attention has been paid to the biological role of arginine and its dietary supplementation in broilers under heat stress (HS) conditions. Therefore, the main aim of this study was to assess the response of broilers to arginine supplementation and cyclic HS, with a focus on liver, pectoral muscle, and blood metabolic profiles and the cecal microbiota. Day-old male Ross 308 broilers (*n* = 240) were placed in 2 rooms with 12 pens each for a 44-day trial. Pens were assigned to one of two groups (6 pens/group/room): the control group (CON) was given a basal diet in mash form and the treated group (ARG) was fed CON diet supplemented with crystalline *L*-arginine. The total arginine:lysine ratio of CON diet ranged between 1.02 and 1.07, while that of ARG diet was 1.20. One room was constantly kept at thermoneutral (TN) conditions, while the birds in the other room were kept at TN conditions until D34 and subjected to cyclic HS from D35 onwards (∼34°C; 9:00 A.M.–6:00 P.M.). Blood, liver, *Pectoralis major* muscle, and cecal content were taken from 2 birds per pen (12 birds/group/room) for metabolomics and microbiota analysis. Growth performance data were also collected on a pen basis. Arginine supplementation failed to reduce the adverse effects of HS on growth performance. Supplemented birds showed increased levels of arginine and creatine in plasma, liver, and *P. major* and methionine in liver, and reduced levels of glutamine in plasma, liver, and *P. major*. HS altered bioenergetic processes (increased levels of AMP and reduced levels of fumarate, succinate, and UDP), protein metabolism (increased protein breakdown to supply the liver with amino acids for energy production), and promoted the accumulation of antioxidant and protective molecules (histidine-containing dipeptides, beta-alanine, and choline), especially in *P. major*. Arginine supplementation may have partially counterbalanced the effects of HS on energy homeostasis by increasing creatine levels and attenuating the increase in AMP levels, particularly in *P. major*. It also significantly reduced cecal observed diversity, while HS increased alpha diversity indices and affected beta diversity. Results of taxonomic analysis at the phylum and family level are also provided.

## 1 Introduction

Global warming is one of the knottiest problems the animal-food industry is and will be facing (Nardone et al., 2010; Bezner Kerr et al., 2022). Rising environmental temperatures have a great impact on the sustainability of chicken meat production, affecting the performance and heath of birds (Renaudeau et al., 2012; Rostagno, 2020) and deteriorating product quality (Song and King, 2015; Wang et al., 2017; Zaboli et al., 2019). The risk of suffering from heat stress (**HS**) and its multifaceted and serious physiological consequences is extremely high for broilers, especially for fast-growing lines (Brugaletta et al., 2022). Looking for strategies intended to prevent or mitigate the detrimental effects of HS is therefore imperative and has become a major research topic in poultry science. Many interventions have been tested so far to help broilers cope with HS, as documented in comprehensive review articles (Gous and Morris, 2005; Lin et al., 2006; Naga Raja Kumari and Narendra Nath, 2018; Saeed et al., 2019; Wasti et al., 2020; Goel, 2021; Nawaz et al., 2021; Vandana et al., 2021).

**FIGURE 1:**
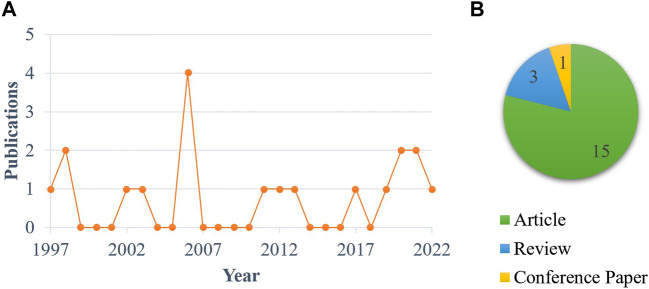
Publication trend **(A)** and type **(B)** of papers mentioning or focusing on arginine in heat-stressed broilers. Note: The literature search was carried out by searching the Scopus database on September 12, 2022 entering the following query string: {TITLE [(chicken OR broiler) AND (arginine OR arg) AND heat AND stress AND NOT (arginine AND vasopressin) AND NOT plant AND NOT polymorphism] OR ABS [(chicken OR broiler) AND (arginine OR arg) AND heat AND stress AND NOT (arginine AND vasopressin) AND NOT plant AND NOT polymorphism]}. No restriction on the date and type of publication was set.

A specific dietary approach has caught our attention, namely formulating broiler diets with levels of arginine, an essential amino acid (**AA**) for chickens (Arnold et al., 1936; Klose et al., 1938; Tamir and Ratner, 1963), above those recommended by the NRC (National Research Council, 1994) or the breeding companies [e.g., Aviagen (2022)]. The reasons for testing arginine supplementation as a potential nutritional strategy for HS alleviation are as follows. First, it has been demonstrated that diet composition and environmental conditions considerably influence arginine requirement of broilers (Khajali and Wideman, 2010; Hassan et al., 2021). In this regard, precise and consistent arginine requirements for broilers under HS are not readily available in the literature. Alterations in feed intake, physiology, metabolism, and gut health and function induced by HS contribute to complicating the calculation of arginine or other nutrient needs for heat-stressed broilers (Teyssier et al., 2022). For example, considering the “ideal protein” concept widely adopted in poultry nutrition (Wiseman and Garnsworthy, 1999), Balnave and Brake (2002) attributed the increased arginine to lysine ratios in broilers undergoing HS to a likely reduction in intestinal absorption of arginine. Second, arginine has been shown to be involved—either directly or through its derivatives—in countless biochemical pathways and body functions, such as modulation of immune, inflammatory, and oxidative responses, regulation of gene expression, protein synthesis, and secretion of anabolic hormones, and contribution to skeletal muscle development, as well as maintenance of gut health, homeostasis, and eubiosis, as recently discussed elsewhere (Brugaletta et al., 2023). Considering these properties, it is worth trying to determine whether high dietary arginine levels produce a reduction in the severity of the effects of HS normally observed in broilers, such as immunodeficiency (Renaudeau et al., 2012; Farag and Alagawany, 2018; Chauhan et al., 2021), inflammation and oxidative stress (Lambert, 2009; Tan et al., 2010; Akbarian et al., 2016; Goel et al., 2021), protein turnover modification indicating catabolic states (Zuo et al., 2015; Lu et al., 2018; Ma et al., 2021), gut health degradation (Rostagno, 2020; Ruff et al., 2020), and perturbation in the gastrointestinal (**GI**) microbiota (Suzuki et al., 1983; Burkholder et al., 2008; Song et al., 2014; He et al., 2021; Liu et al., 2022). Third, the significant arginine-mediated improvements in performance found in broiler studies conducted under thermoneutral (**TN**) settings (Basoo et al., 2012; Xu et al., 2018; Zampiga et al., 2018; Liu et al., 2019; Sirathonpong et al., 2019; Brugaletta et al., 2023) make arginine supplementation a promising tool for minimizing performance losses caused by HS. Nevertheless, and this is the fourth and final reason, very little attention has been paid to the biological role of arginine and its dietary supplementation in heat-stressed broilers (Figure 1), suggesting that there is much room for further progress in these intriguing research areas. An essential step to take is to clarify the role of arginine in metabolism and intestinal health of broilers, which are profoundly affected by HS, as mentioned above.

Therefore, the main aim of this study was to assess the response of broilers to arginine supplementation and HS, with a focus on liver, pectoral muscle, and blood metabolic profiles and the cecal microbiota. Specifically, control and supplemented birds were subjected to a cyclic HS period to simulate field conditions typical of the summer in temperate areas of the world, that is a succession of diurnal hot temperatures and cooler nights. Growth performance data were also collected and analyzed to get as complete a picture as possible.

## 2 Materials and methods

### 2.1 Experimental design, housing, and husbandry conditions

In this study, approved by the Ethical Committee of the University of Bologna (ID: 4387), birds were reared, monitored, and slaughtered in compliance with EU legislation (i.e., Dir. 2007/43/EC, Reg. 2009/1099/EC, and Dir. 2010/63/EU) and had *ad libitum* access to feed and water.

**FIGURE 2:**
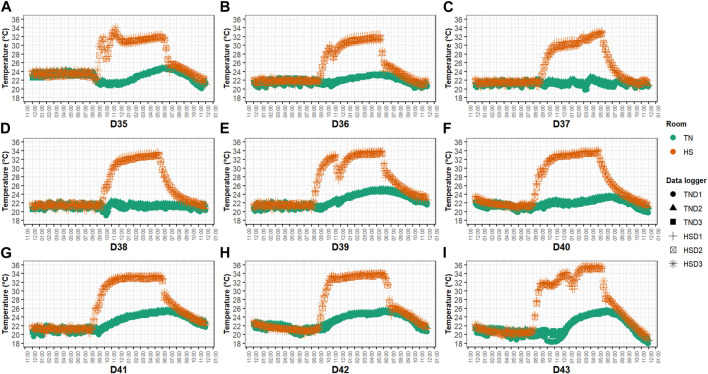
Hourly temperature **(A–I)** of the two rooms during the cyclic HS period. Note: D#, day#; TN, thermoneutral conditions; HS, (cyclic) heat stress conditions; TND#, data logger# in room TN; HSD#, data logger# in room HS.

One-day-old male Ross 308 broilers (*n* = 240) were supplied by a commercial hatchery and vaccinated against infectious bronchitis, Marek’s, Newcastle and Gumboro diseases, and coccidiosis. Chicks were randomly placed in 2 identical environmental chambers (Bologna, Italy), hereafter referred to as rooms. Each room was divided into 12 equally sized floor pens equipped with ∼3 kg/m^2^ of chopped straw as bedding material (litter of ∼7 cm in depth), a bell feeder (minimum 4 cm of feeder front space per bird), and 1 nipple drinker for every 3 birds. Pens were randomly assigned to one of two experimental groups (i.e., 6 replicate pens/group/room) and were arranged in a block design. The control group (**CON**) was given a commercial antibiotic-free basal diet in mash form, which was formulated to meet the nutrition specifications released by the breeding company (Aviagen, 2022). The treated group (**ARG**) was fed the same basal diet supplemented on top with crystalline *L*-arginine (∼1.5 g/kg feed; purity of 98%; BESTAMINO™, CJ BIO, Seoul, Korea). The basal diet formula and composition according to the three-phase feeding program used (i.e., starter, 0–14 days; grower, 15–27 days; finisher, 28–44 days) are shown in Table 1. Analysis of AA concentration of the experimental diets was outsourced to Evonik Industries AG labs (Hanau, Germany). The total arginine level of the basal diet was 1.50, 1.38, and 1.23%, while that of the supplemented diet was 1.74, 1.56, and 1.37% in the starter, grower, and finisher phase, respectively. The total arginine to lysine ratio of the control diet ranged between 1.02 and 1.07 and was consistent with the breeding company’s specifications (Aviagen, 2022), whereas that of the supplemented diet was 1.20 in all feeding phases. The artificial photoperiod was 23 L:1D during the first 7 and last 3 days, while 18 L:6D (i.e., light phase 6:00 A.M.–10:00 P.M., dark phase 10:00 P.M.–02:00 A.M., light phase 02:00 AM–04:00 AM, and dark phase 04:00 AM–06:00 AM) for the remainder days following EU legislation (i.e., Dir. 2007/43/EC) and the breeding company’s guidelines for lightning and pre-processing management (Aviagen, 2018). Environmental temperature and relative humidity (**RH**) were recorded with climate data loggers (Trotec GmbH, Heinsberg, Germany) located at animal level (3 data loggers/room having a recording time of 900 s). As for the temperature program, one room was constantly kept at TN conditions following the instructions of the breeding company (Aviagen, 2018), while the birds in the other room were kept at TN conditions until D34 and subjected to cyclic HS from D35 to D43, with the temperature increased daily to ∼34°C from 9:00 A.M. to 6:00 P.M. (Figure 2). RH was adjusted by means of humidifiers (Trotec GmbH) and it ranged between 40% and 70% in both rooms during the HS period.

**TABLE 1:** Basal diet formula and composition according to the 3-phase feeding program.

Ingredient (g/100 g feed)	Feeding phase		
	Starter (0–14 days)	Grower (15–27 days)	Finisher (28–44 days)
Corn	29.87	32.49	15.02
White corn	0.00	0.00	6.00
Wheat	24.97	24.97	39.95
Pea	3.00	3.00	3.00
Soybean meal	15.89	15.17	8.68
Roasted soybean	9.99	14.99	16.99
Potato protein meal	3.51	0.00	0.00
Sunflower meal	3.00	3.00	3.00
Corn gluten meal	3.00	0.00	0.00
Soybean oil	2.27	2.83	4.47
Calcium carbonate	0.19	0.56	0.87
Dicalcium phosphate	1.54	0.48	0.00
Sodium bicarbonate	0.05	0.00	0.05
Sodium chloride	0.31	0.33	0.27
Choline chloride	0.10	0.10	0.06
Lysine sulphate	0.67	0.63	0.54
*DL*-Methionine	0.30	0.33	0.29
*L*-Threonine	0.14	0.18	0.14
Amino acid mix (arginine, valine, isoleucine)	0.35	0.24	0.17
Non-starch polysaccharides-degrading enzyme	0.05	0.05	0.05
Phytase	0.20	0.20	0.20
Mycotoxin binder	0.10	0.00	0.00
Vitamin and mineral premix^a^	0.50	0.45	0.25
**Composition**			
Dry matter^b^ (%)	88.24	88.28	88.52
Crude protein^b^ (%)	22.88	20.06	18.36
Total lipid^b^ (%)	5.89	7.38	9.18
Crude fiber^b^ (%)	3.05	3.18	3.11
Ash^b^ (%)	5.00	4.47	4.06
Total lysine^b^ (%)	1.47	1.31	1.15
Total arginine^b^ (%)	1.50	1.38	1.23
Total arginine: lysine^b^	1.05	1.07	1.09
Total methionine + cysteine^b^ (%)	1.08	0.99	0.88
Calcium (%)	0.78	0.65	0.59
Phosphorus (%)	0.63	0.46	0.36
Metabolizable energy (kcal/kg)	3,072	3,146	3,296

^a^ The premix provides the following per kg of feed: Vitamin A (retinyl acetate), 12,500 IU; vitamin D3, 5,000 IU (i.e., cholecalciferol, 3,500 IU + 25-OH, D3, 1,500 IU); Vitamin E (*DL*-α-tocopheryl acetate), 125 mg; Vitamin K (menadione sodium bisulfite), 6.75 mg; riboflavin, 9.0 mg; Pantothenic acid, 22.0 mg; niacin, 75 mg; Pyridoxine, 5 mg; folic acid, 3.0 mg; Biotin, 0.35 mg; Thiamine, 4.0 mg; Vitamin B_12_, 50 μg; Mn, 100 mg; Zn, 102 mg; Fe, 30 mg; Cu, 15 mg; I, 2.0 mg; Se, 0.35 mg.

^b^ Analyzed values.

### 2.2 Data and sample collection

Growth performance data were collected on a replicate basis. The number and body weight (**BW**) of birds were recorded at placement (D0), at every feeding phase switch (D15/28), and at slaughter (D44), while feed intake (**FI**) was measured for each feeding phase. Daily weight gain (**DWG**), daily feed intake (**DFI**), and feed conversion ratio (**FCR**) were calculated for the feeding phases separately and cumulative FI and cumulative FCR were computed for the period consisting of a feeding phase and its previous one/ones (i.e., starter + grower; starter + grower + finisher). The number and BW of dead or culled birds were daily recorded daily to compute the mortality rate and correct the performance data for mortality.

Three birds per pen (i.e., 18 birds/group/room) were randomly chosen and labeled to measure the rectal temperature with a veterinary thermometer (Scala Electronic GmbH, Stahnsdorf, Germany). The rectal temperature was taken on the first and eighth day of the HS period (i.e., D35 and D42, respectively) at two time points, namely 9:00 A.M. and 6:00 P.M.

At slaughter in a commercial abattoir (D44), biological samples were collected from two of the three birds previously labeled in each pen (i.e., 12 birds sampled/group/room). Blood was taken from the wing vein and kept at RT before being centrifuged to get plasma, which was subsequently stored at −80°C until metabolomics analysis through proton nuclear magnetic resonance (^**1**^ **H-NMR**). Hepatic tissue (∼1 cm^3^) was dissected from the right caudal lobe of the liver, frozen in liquid N_2_, and stored at −80°C until ^1^H-NMR analysis. Breast muscle (∼1 cm^3^) was taken from the left cranial portion of the *Pectoralis major*, frozen in liquid N_2_, and stored at −80°C until ^1^H-NMR analysis. Lastly, the content of both ceca was collected, frozen in liquid N_2_, and stored at −80°C until DNA extraction for shotgun metagenomic sequencing.

### 2.3 Lab analysis

For metabolomics analysis, an ^1^H-NMR solution with D_2_O, containing TSP 10 mmol/L and NaN_3_ 2 mmol/L, was created. Phosphate buffer 1 M was used to achieve a pH of 7.00 ± 0.02, while TSP was used as a reference for NMR chemical-shift and NaN3 avoided bacterial proliferation. Approximately 0.5 g of liver and muscle samples were homogenized (14,000 rpm; 20 s; RT) with 3 mL of a water solution of TCA 7% (w/w). All samples were centrifuged (18,630 g; 900 s; 4°C) and 0.7 mL of supernatant were mixed with 0.1 mL of the ^1^H-NMR solution. The pH of liver and muscle samples was further adjusted to 7.00 ± 0.02 with drops of NaOH 9 N and 1 N as needed. All samples were centrifuged again at the abovementioned conditions. The ^1^H-NMR spectra were registered (600.13 MHz; 298 K) with an AVANCE™ III spectrometer (Bruker, Milan, Italy) equipped with TopSpin software v3.5 (Bruker). The signals from broad resonances due to large molecules were suppressed with CPMG-filter (400 echoes with a *t* of 400 µs and a 180°pulse of 24 µs, for a total filter of 330 ms), while the residual signal of water was suppressed by means of presaturation. This was done employing the cpmgpr1d sequence, part of the standard pulse sequence library. Each spectrum was acquired summing up 256 transients constituted by 32,000 data points encompassing a window of 7,184 Hz, separated by a relaxation delay of 5 s. The ^1^H-NMR spectra were phase-adjusted in TopSpin v3.5 (Bruker) and then exported to ASCII format by means of the built-in script convbin2asc. Spectra were processed with R (R Core Team, 2020) through home-made scripts. Signal assignment was performed comparing their chemical shift and multiplicity with Human Metabolome Database (Wishart et al., 2007) and Chenomx software library v10 (Chenomx Inc. Edmonton, Canada), by means of Chenomx software routines. For all samples, the absolute concentration of molecules was performed with the median water dilution, assessed *via* probabilistic quotient normalization (Dieterle et al., 2006). TSP was used as an internal standard. Differences in water content between samples from the same matrix were considered through probabilistic quotient normalization. The concentration of each molecule was obtained from the area of one of its signals, calculated by the global spectra deconvolution algorithm implemented in MestReNova software v14.2.0–26256 (Mestrelab research S.L. Santiago De Compostela, Spain), by considering a limit of quantification of 5. This was done after applying a baseline adjustment by Whittaker Smoother procedure and a line broadening of 0.3.

Moving to microbiota analysis of the cecal content, DNA extraction was performed adopting a bead-beating procedure and using the QIAmp^®^ DNA Stool Mini Kit (Qiagen, Milan, Italy). Total DNA was fragmented and tagged with sequencing indexes and adapters using the Nextera XT DNA Library Preparation Kit (Illumina, San Diego, CA, United States.). Shotgun metagenomic sequencing was performed with NextSeq 500 (Illumina) 2 × 149 bp in paired-end mode. Filtering of low-quality reads and sequence adapters trimming of raw reads were conducted using the tool AdapterRemoval. The microbial community composition was evaluated with the bioinformatic tool MetaphlAn3 (Truong et al., 2015) at the phylum level. Alpha diversity indices (i.e., observed, Shannon, Simpson, and Inverse Simpson) and Bray-Curtis beta distance were calculated with the R package *phyloseq* (McMurdie and Holmes, 2013).

### 2.4 Data analysis

The effect of the factor group on growth performance of the starter and grower phases was assessed with a two-way blocked ANOVA without interaction, considering the room as a fixed factor and using the replicate pen as the experimental unit. The data from the finisher phase, however, were analyzed as a 2 × 2 factorial design using a two-way blocked ANOVA with interaction between the main factors group and room. Tukey’s HSD *post hoc* test was used if needed. Mortality rate data were transformed using the arcsine transformation before being analyzed with inferential statistics.

Rectal temperature data were grouped by the day of collection and analyzed through a three-way mixed ANOVA, a type of repeated-measures ANOVA that includes between-subject factors (i.e., group and room) and within-subject factors (i.e., time point). After verifying that there was no statistically significant three-way interaction, rectal temperature data were grouped by the factors group and room to run paired *t*-tests with Bonferroni adjustment between time points. The measured bird represented the experimental unit for rectal temperature data analysis.

Regarding the analysis of metabolomics data, a two-way ANOVA with interaction between group and room and the sampled bird as the experimental unit was carried out. Tukey’s HSD *post hoc* test was used where appropriate. Metabolomics data deviating from normality in Shapiro-Wilk test were subjected to Box-Cox transformation (Box and Cox, 1964).

The effects of group, room, and their interaction on alpha diversity measures and beta diversity distance matrix were tested with a two-way ANOVA and a two-way PERMANOVA with interaction between group and room [adonis2 function implemented in the R *vegan* package (Oksanen et al., 2020)], respectively. Similarly, relative abundance data at the phylum and family level were analyzed with a two-way ANOVA followed by a Tukey’s HSD *post hoc* test where appropriate. The sampled bird was used as the experimental unit for the statistical analysis of diversity measures and relative abundance data.

These analyses were carried out using R (R Core Team, 2020). *p*-values less than 0.05 were considered significant, while those ranging between 0.05 and 0.1 were considered tendencies.

## 3 Results

Growth performance results are shown in Table 2. Chicks did not show significantly different weights between groups at placement. Before HS was applied, ARG birds tended to be heavier than CON birds at the end of the starter phase (i.e., BW of 450.4 vs. 437.9; *p* = 0.09). In contrast, FCR of group ARG was significantly lower than that of group CON (1.270 vs. 1.344; *p* = 0.02). Similarly, at the end of the grower phase, ARG birds showed greater BW and lower cumulative FCR than CON birds (1,471 g vs. 1,424 g and 1.553 vs. 1.646; *p* < 0.05). The difference in DWG, however, approached but did not achieve significance (77.95 vs. 75.16 g; *p* = 0.08). In the finisher phase, neither the effect of the factor group nor that of the interaction between group and room was significant. The exposure to HS, however, decreased (*p* ≤ 0.01) BW, DWG, DFI, and (cumulative) FI, while increased (*p* < 0.05) mortality regardless of the group.

**TABLE 2:** Growth performance of groups CON and ARG in the three-phase feeding trial.

Feeding phase	Factor	Dependent variable								
		Chick weight (g/bird)	BW (g/bird)	DWG^a^ (g/bird/d)	DFI^a^ (g/bird/d)	FI^a^ (kg/bird)	Cum. FI^a^ ^,b^ (kg/bird)	FCR^a^	Cum. FCR^a^ ^,b^	Mortality (%)
**Starter** ^**c**^ **(0–14 days)**	**Group**									
	CON	43.15	437.9	28.20	37.94	0.531	—	1.344	—	0.00
	ARG	43.60	450.4	29.06	36.91	0.517	—	1.270	—	0.00
	**SE**	1.07	16.69	1.22	2.28	0.03	—	0.06	—	0.00
	*p* **-value**	0.327	0.094	0.113	0.292	0.292	—	**0.015**	—	—
**Grower** ^**c**^ **(15–27 days)**	**Group**									
	CON	—	1,424	75.16	133.1	1.730	2.261	1.772	1.646	0.83
	ARG	—	1,471	77.95	130.0	1.690	2.207	1.670	1.553	0.00
	**SE**	—	51.59	3.53	9.77	0.13	0.12	0.15	0.10	0.07
	*p* **-value**	—	**0.049**	0.079	0.459	0.459	0.295	0.112	**0.042**	0.339
**Finisher** ^**d**^ **(28–44 days; cyclic HS from D35 onwards)**	**Group**									
	CON	—	3,051	96.51	196.0	3.332	5.592	2.033	1.852	5.00
	ARG	—	3,138	98.21	188.5	3.205	5.412	1.927	1.751	0.83
	**Room**									
	TN	—	3,222	105.1	208.3	3.541	5.743	1.987	1.811	0.00
	HS	—	2,967	89.64	176.3	2.996	5.261	1.973	1.792	5.83
	**Group × Room**									
	CON-TN	—	3,188	104.1	217.4	3.696	5.939	2.096	1.894	0.00
	ARG-TN	—	3,257	106.1	199.1	3.385	5.546	1.878	1.728	0.00
	CON-HS	—	2,914	88.93	174.5	2.967	5.246	1.970	1.810	10.00
	ARG-HS	—	3,019	90.35	178.0	3.026	5.277	1.975	1.775	1.67
	**SE**	—	122.0	4.78	15.47	0.26	0.37	0.20	0.15	0.14
	*p* **-value**									
	Group	—	0.111	0.404	0.266	0.266	0.259	0.229	0.122	0.179
	Room	—	**< 0.001**	**< 0.001**	**< 0.001**	**< 0.001**	**0.010**	0.866	0.759	**0.039**
	Group × Room	—	0.732	0.889	0.115	0.115	0.191	0.208	0.297	0.179

^a^Corrected for mortality.

^b^ Computed for the period consisting of a feeding phase and its previous one/ones.

^c^ *n* = 12 replicate pens/group.

^d^ *n* = 6 replicate pens/group/room. In “room HS”, temperature was raised daily to ∼34°C from 9:00 A.M. to 6:00 P.M. from D35 to D43.

Note: *p*-values less than 0.05 are in bold. BW, body weight; DWG, daily weight gain; DFI, daily feed intake; FI, feed intake; FCR, feed conversion ratio; Cum., cumulative; CON, control group; ARG, arginine-supplemented group; SE, standard error; TN, thermoneutral conditions; HS, (cyclic) heat stress conditions.

**FIGURE 3:**
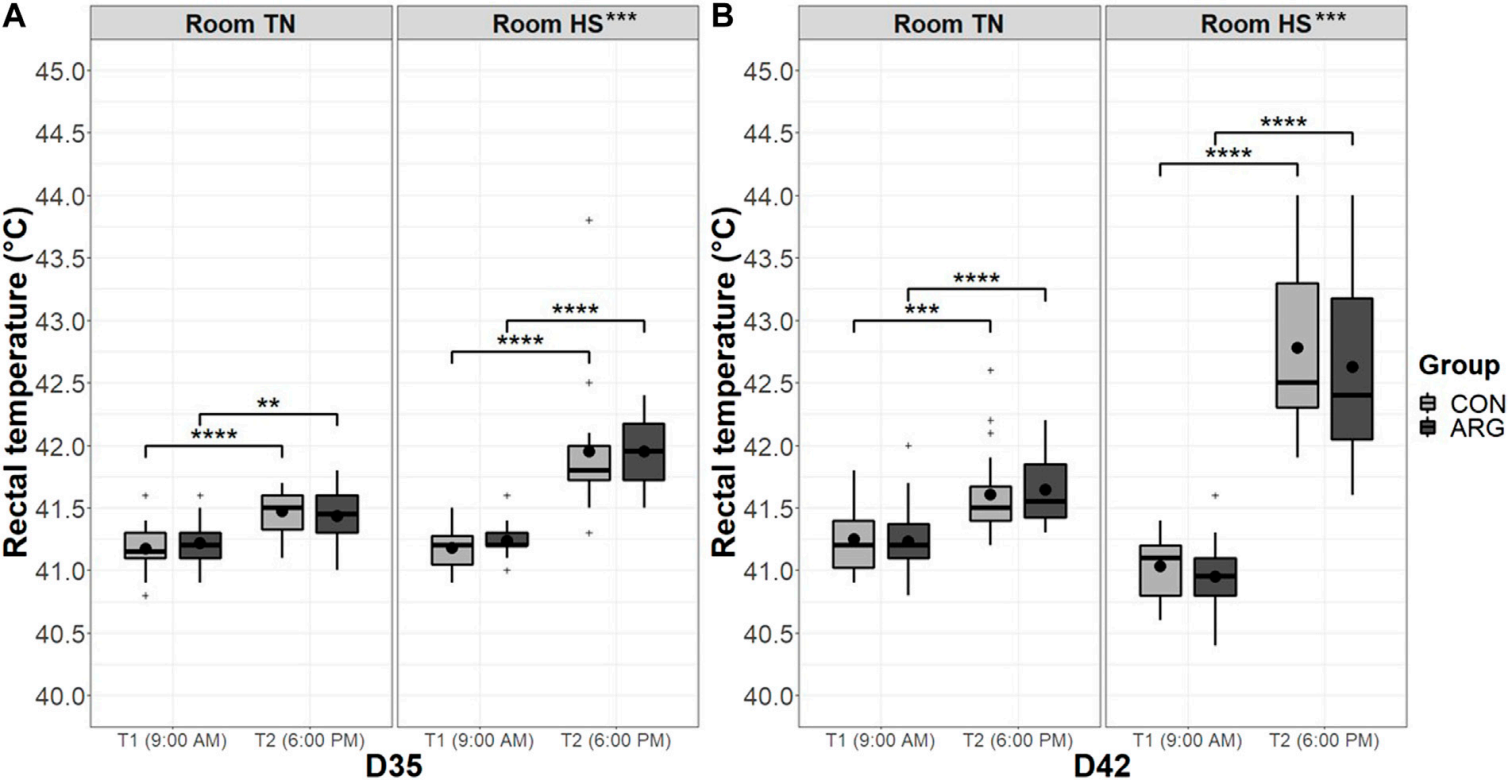
Rectal temperature of CON and ARG birds in the two rooms on the first **(A)** and eighth **(B)** day of the cyclic HS period at two time points. Note: The rectal temperature of 18 labeled birds/group/room was measured on the first (D35) and the eighth (D42) day of the cyclic HS period at two time points, namely T1 (9:00 AM) and T2 (6:00 P.M.). Group means are the black dots inside the boxes. **, *p* < 0.01; ***, *p* < 0.001; ****, *p* < 0.0001. TN, thermoneutral conditions; HS, (cyclic) heat stress conditions; T1/2, time point 1/2; D#, day#; CON, control group; ARG, arginine-supplemented group.

The rectal temperature of representative birds of groups CON and ARG on the first and eighth day of the cyclic HS period at 9:00 A.M. and 6:00 P.M. is presented in Figure 3. The raw data ranged between 40.4°C and 44.0°C. The mixed ANOVA used to analyze the data grouped by the day of collection did not reveal any significant first-order interaction between the three factors tested (i.e., group, room, and time point). Moreover, the effect of the factor group was not significant, while those of the factors room, time point, and their interaction were highly significant (*p* < 0.001). The pairwise comparisons of time points indicated that the increase in rectal temperature from 9:00 A.M. to 6:00 P.M. was greatly significant (*p* < 0.001) in either room, on both the measurement days, and irrespective of the group.

The concentration of several metabolites was found to be affected by the factors group and room in plasma, liver, and *P. major*, as shown in Supplementary Tables S1–S3. The data of these tables have been compared and summarized in Table 3 focusing on potentially biologically relevant metabolites. Group ARG had a higher concentration of arginine in plasma, liver (*p* < 0.001), and *P*. *major* (*p* = 0.07), creatine in plasma, liver and *P*. *major* (*p* < 0.05), methionine in liver (*p* = 0.08), glycerol and lactate in *P*. *major* (*p* < 0.05), as well as a lower concentration of alanine in plasma (*p* < 0.05) and *P*. *major* (*p* = 0.08), AMP in liver and *P*. *major* (*p* < 0.05), glutamine in plasma (*p* = 0.01), liver (*p* = 0.06), and *P*. *major* (*p* = 0.1), glutathione in liver and *P*. *major* (*p* ≤ 0.01), and glucose-1-phosphate in *P*. *major* (*p* = 0.07). The exposure to HS increased the concentration of glucose in plasma (*p* < 0.05), phenylalanine in plasma and liver (*p* < 0.05), glutamate (*p* < 0.05), (iso)leucine (*p* = 0.07), threonine (*p* = 0.05), uracil and uridine (*p* < 0.05) in liver, AMP (*p* < 0.05), anserine, beta-alanine, carnosine (*p* ≤ 0.001), and choline (*p* = 0.01) in *P*. *major*, while decreased that of aspartate, betaine, carnosine, glycerol, and methionine in plasma (*p* < 0.05), succinate in plasma and liver (*p* < 0.05), glycine (*p* < 0.01) and tyrosine (*p* ≤ 0.09) in plasma and *P*. *major*, UDP in liver and *P*. *major* (*p* < 0.01), alanine, fumarate, and N,N-Dimethylglycine in plasma, liver and *P*. *major* (*p* ≤ 0.05), and (iso)leucine, threonine, and valine in *P*. *major* (*p* ≤ 0.002). On the other hand, the interactive effect of the factors group and room tended to be or was significant on the concentration of a few metabolites, such as carnosine and formate in plasma, sarcosine in plasma and liver, ethanolamine and propylene glycol in liver, and isoleucine and threonine in *P*. *major*.

**TABLE 3:** Biologically relevant metabolites whose concentration was affected (*p*-values of 0.1 maximum) by either arginine supplementation, HS exposure or both (see Supplementary Tables S1–S3 for more information).

Metabolite	Arginine supplementation^a^			HS exposure^a^		
	Plasma	Liver	*P. major*	Plasma	Liver	*P. major*
Alanine	↓	-	↓	↓	↓	↓
AMP	-	↓	↓	-	-	↑
Anserine	-	-	-	-	-	↑
Arginine	↑	↑	↑	-	-	-
Aspartate	-	-	-	↓	-	-
Beta-alanine	-	-	-	-	-	↑
Betaine	-	-	-	↓	-	-
Carnosine	-	-	-	↓	-	↑
Choline	-	-	-	-	-	↑
Creatine	↑	↑	↑	-	-	-
Fumarate	-	-	-	↓	↓	↓
Glucose	-	-	-	↑	-	-
Glucose-1-phosphate	-	-	↓	-	-	-
Glutamate	-	-	-	-	↑	-
Glutamine	↓	↓	↓	-	-	-
Glutathione	-	↓	↓	-	-	-
Glycerol	-	-	↑	↓	-	-
Glycine	-	-	-	↓	-	↓
Isoleucine	-	-	-	-	↑	↓
Lactate	-	-	↑	-	-	-
Leucine	-	-	-	-	↑	↓
Methionine	-	↑	-	↓	-	-
N, N-Dimethylglycine	-	-	-	↓	↓	↓
Phenylalanine	-	-	-	↑	↑	-
Succinate	-	-	-	↓	↓	-
Threonine	-	-	-	-	↑	↓
Tyrosine	-	-	-	↓	-	↓
UDP	-	-	-	-	↓	↓
Uracil	-	-	-	-	↑	-
Uridine	-	-	-	-	↑	-
Valine	-	-	-	-	-	↓

^a^ Compared with concentrations measured in the samples collected from birds fed the control diet or reared under TN conditions, the symbols indicate: ↑, increased; ↓, decreased; -, not affected.

As for the results of the cecal microbiota analysis, it can be seen from Table 4 that group ARG showed reduced observed diversity (*p* < 0.05) compared to group CON, while HS increased this alpha index (*p* = 0.09), as well as the Shannon (*p* < 0.01) and Inverse Simpson (*p* = 0.05). No significant interaction between group and room was found for alpha diversity indices. Beta diversity was also affected by group (*p* = 0.001) and room (*p* = 0.07), but not by their interaction (*p* = 0.260). Differences in relative abundance of important cecal bacterial phyla and families for poultry are reported in Table 5 (see Supplementary Table S4 for more information). The data indicate that ARG birds harbored less (*p* < 0.01) Actinobacteria than CON birds, while the other phyla were not significantly affected by the factor group. However, room had a significant but opposite effect on Bacteroidetes and Firmicutes, with the former showing a lower (*p* = 0.08) abundance and the latter a higher (*p* < 0.05) abundance in the ceca of HS birds than those of TN birds. Moving to bacterial families, Bacilli unclassified significantly increased due to arginine supplementation and showed the greatest (*p* = 0.001) abundance in the ceca of ARG birds. Nevertheless, the effect of the factor room seems to prevail, as Enterobacteriaceae, Enterococcaceae, and Lachnospiraceae all increased due to HS exposure (*p*-values ranging from 0.07 to 0.02). An interactive effect between the factors group and room on the abundance of Bacteroidaceae (*p* = 0.07), Lachnospiraceae (*p* = 0.09), and Lactobacillaceae (*p* < 0.01) was found. The separation of the means showed that CON birds subjected to HS had more (*p* < 0.05) Lactobacillaceae than the other birds.

**TABLE 4:** Alpha diversity indices of the cecal content of CON and ARG birds in the two rooms at D44.

Factor	Alpha diversity index			
	Observed	Shannon	Simpson	Inverse simpson
Group				
CON	189	3.65	0.94	19.1
ARG	180	3.60	0.95	19.3
**Room**				
TN	181	3.56	0.94	17.9
HS	189	3.70	0.95	20.5
**Group × Room**				
CON-TN	184	3.57	0.94	17.5
ARG-TN	177	3.54	0.94	18.3
CON-HS	194	3.72	0.95	20.6
ARG-HS	183	3.66	0.95	20.3
**SE**	15.64	0.17	0.02	4.51
*p* **-value**				
Group	**0.049**	0.403	0.802	0.855
Room	0.085	**0.007**	0.100	0.052
Group × Room	0.589	0.698	0.589	0.667

Note: *n* = 12 birds/group/room. *p*-values less than 0.05 are in bold. CON, control group; ARG, arginine-supplemented group; TN, thermoneutral conditions; HS, (cyclic) heat stress conditions; SE, standard error.

**TABLE 5:** Relative abundances (%) of important bacterial phyla and families in the cecal content of CON and ARG birds in the two rooms at D44.

Factor	Phylum				Family									
	Actinobacteria	Bacteroidetes	Firmicutes	Proteobacteria	Bacilli unclassified	Bacteroidaceae	Clostridia unclassified	Clostridiaceae	Enterobacteriaceae	Enterococcaceae	Lachnospiraceae	Lactobacillaceae		Ruminococcaceae
Group														
CON	1.5	6.8	89.3	1.8	0.2	0.8	17.9	2.3	0.6	0.05	8.5		0.9	36.9
ARG	0.8	7.6	88.0	1.5	0.7	0.8	17.5	1.9	0.4	0.10	8.5		0.5	36.1
**Room**														
TN	1.2	8.3	86.7	1.6	0.4	0.8	17.1	2.2	0.4	0.02	7.9		0.3	37.2
HS	1.1	6.2	90.6	1.7	0.5	0.7	18.3	2.0	0.7	0.12	9.1		1.1	35.8
**Group × Room**														
CON-TN	1.4	8.1	88.1	1.6	0.2	0.6	17.5	2.6	0.4	0.001	8.4		0.2 b	37.8
ARG-TN	0.9	8.5	85.4	1.6	0.6	1.1	16.7	1.8	0.4	0.05	7.4		0.4 b	36.6
CON-HS	1.7	5.6	90.5	1.9	0.3	1.0	18.2	2.0	0.9	0.10	8.5		1.6 a	36.0
ARG-HS	0.6	6.8	90.7	1.4	0.8	0.5	18.4	2.0	0.5	0.15	9.7		0.6 b	35.7
*p* **-value**														
Group	**0.008**	0.495	0.461	0.216	**0.001**	0.979	0.863	0.160	0.234	0.234	0.897		**0.048**	0.654
Room	0.985	0.081	**0.027**	0.730	0.275	0.716	0.486	0.484	0.070	**0.024**	0.064		**< 0.001**	0.431
Group × Room	0.254	0.770	0.390	0.323	0.617	0.072	0.767	0.176	0.226	0.956	0.089		**0.004**	0.778

Note: *n* = 12 birds/group/room. *p*-values less than 0.05 are in bold. Means that fall under the interaction between group and room and show distinct letters are significantly different (*p* < 0.05). CON, control group; ARG, arginine-supplemented group; TN, thermoneutral conditions; HS, (cyclic) heat stress conditions; SE, standard error.

## 4 Discussion

While secondary to the main objective of the study, it is worth opening this discussion with an examination of growth performance results. Before the application of HS, arginine supplementation significantly reduced FCR (−5.7%) and increased BW at D27 (+3.3%), which accord with our earlier observations (Zampiga et al., 2018; Brugaletta et al., 2023) and those reported by other authors (Basoo et al., 2012; Xu et al., 2018; Liu et al., 2019; Sirathonpong et al., 2019). This therefore confirms that feeding diets high in arginine improves the performance of broilers under TN conditions. On the other hand, cyclic exposure to high environmental temperatures considerably impaired growth performance, reducing FI and BW gain, and increased body temperature and mortality, thereby causing some of the adverse effects typically observed in heat-stressed chickens (Brugaletta et al., 2022; Teyssier et al., 2022). Arginine supplementation, however, did not alleviate the deterioration in performance or prevent environmentally induced hyperthermia, as the birds responded similarly to the thermal stress irrespective of the diet they were fed on. While performance and body temperature results are reliable because they are based on a fair number of replicates and rectal temperature measurements, respectively, speculations about mortality data must be made cautiously due to the modest number of birds per pen, which has made mortality rate a variable very susceptible to change in this trial. Data from the literature suggest that arginine supplementation could help broilers counteract the detrimental effects of HS on growth performance (Hassan et al., 2021; Teyssier et al., 2022). Nevertheless, our results seem to contradict that performance of heat-stressed broilers benefits from this nutritional strategy. A likely explanation for this inconsistency is that the favorable effects of arginine supplementation may have been somewhat blunted by the substantial reduction in FI caused by HS, which resulted in the ingestion of insufficient dietary arginine to preserve performance. It should be noted, however, that the reduction in FI was not of the same magnitude for control (−19.7%) and supplemented (−10.7%) birds under HS compared to their TN counterparts. Testing greater levels of dietary arginine to offset the FI loss might therefore be advantageous if further investigations on arginine supplementation for heat-stressed broilers are to be undertaken.

Anyway, the chief goal of the current study was to evaluate the metabolic and microbiota response of broilers to arginine supplementation and HS. Supplemented birds showed a significantly higher concentration of arginine in plasma—supporting again the results formerly reported by our lab (Zampiga et al., 2018; Brugaletta et al., 2023), as well as by Kidd et al. (2001) and investigators working with piglets (Kim et al., 2004) and rats (Holecek and Sispera, 2016)—liver, and *P. major*. These results provide further support for the hypothesis that increasing dietary arginine levels beyond those recommended leads to a considerable increase in its bioavailability, which is of paramount importance for animals unable to synthesize arginine *de novo*, such as chickens (Ball et al., 2007).

Metabolomics analyses also revealed greater concentrations of hepatic and circulating creatine for arginine-supplemented birds, reflecting what we have previously found (Brugaletta et al., 2023), as well as higher levels of creatine in *P. major*. The latter result confirms the observation of Chamruspollert et al. (2002) that breast muscles of broilers fed diets supplemented with arginine showed increased creatine content. Creatine, an AA derivative at the base of which are arginine and glycine, is produced largely in the liver and then transported to target tissues by blood (Walker, 1960; Oviedo-Rondón and Córdova-Noboa, 2020), serving, for example, as a key metabolite for skeletal muscle function and energy homeostasis (Oviedo-Rondón and Córdova-Noboa, 2020). Interestingly, it has been shown that dietary arginine intake affects creatine levels in different parts of the chicken’s body (Keshavarz and Fuller, 1971a; 1971b; Chamruspollert et al., 2002) and that creatine supplementation improves growth performance and breast meat yield of broilers (Ringel et al., 2007; Oviedo-Rondón and Córdova-Noboa, 2020; Portocarero and Braun, 2021). In light of this, the increase in creatine levels can be considered a positive outcome of the arginine supplementation tested here.

Histidine-containing dipeptides and betaine were affected by HS exposure. Specifically, heat-stressed birds showed increased concentrations of carnosine and anserine in *P. major*, which is consistent with our earlier findings (Zampiga et al., 2021), while reduced concentrations of carnosine and betaine in plasma. Furthermore, HS raised the levels of beta-alanine and choline in *P. major*. Carnosine consists of histidine and beta-alanine, while anserine is formed by methylation of carnosine. These two dipeptides have been shown to have potent antioxidant activity and several biological functions, such as pH buffering, metal-ion chelation, complexing of dangerous carbonyl compounds, and anticross-linking effect on proteins, thereby protecting cells against stress and ischemia, as recently reviewed by Lackner et al. (2021). Betaine has been found to improve health, performance, carcass composition, and meat quality of poultry (Metzler-Zebeli et al., 2009). Being a powerful osmolyte, betaine can mitigate the effects of HS on cells (Ratriyanto and Mosenthin, 2018). Choline is an important provider of methyl groups and its metabolism is intimately related to that of betaine (a product of choline oxidation) and methionine. Choline is a multifunctional molecule that, among other things, constitutes many phospholipids (e.g., phosphatidylcholine) that maintain integrity and functions of cell membranes (Simon, 1999). Taken together, our present and previous data (Zampiga et al., 2021) support the idea that the accumulation of antioxidant and protective molecules in specific tissues, such as the pectoral muscle, is an essential part of the adaptive response to HS in chickens.

Besides arginine, the liver of arginine-supplemented birds was enriched in methionine, the first limiting AA for chickens (Leeson and Summers, 2001). This finding can be interpreted as a confirmation of what was previously assumed, namely that an improvement in intestinal mucosal health, integrity, function, and morphology mediated by arginine leads to improved digestion and absorption of dietary AAs (Brugaletta et al., 2023). Feeding the diet supplemented with arginine also resulted in reduced levels of the conditionally essential AA glutamine in the blood, similarly to what was observed in our former investigation (Brugaletta et al., 2023), as well as in liver, and *P. major*. Glutamine has been shown to be extremely important in supporting GI tract development and function and promoting gut health (Bortoluzzi et al., 2018; 2020). Its dietary supplementation has been found to attenuate the negative effects of enteric challenges in broilers, such as those of necrotic enteritis, coccidiosis, and *Salmonella* infection (Xue et al., 2018; Oxford and Selvaraj, 2019; Wu et al., 2022), as well as the impacts of HS on intestinal barrier integrity in mice as discussed in the review article by Bortoluzzi et al. (2018). Coster et al. (2004) pointed out that most of dietary glutamine and a quarter of glutamine in the blood are used by enterocytes and intestinal immune cells as a vital nutrient to obtain nitrogen and energy. Consequently, there is a real competition between the gut and extraintestinal tissues for glutamine. These authors also reported that almost all plasma glutamine is derived from the pool of free glutamine in skeletal muscle, the largest reservoir and most important site for the synthesis of this AA. In the liver, however, glutamine is utilized as a substrate for gluconeogenesis and the synthesis of urea (in mammals), acute phase proteins, and glutathione. The reasons for the reduction in glutamine levels observed in the present study remain to be defined, but it can be hypothesized that intestinal mucosa of arginine-supplemented birds had an increased demand for glutamine, thereby draining arterial glutamine to fuel the accelerated metabolism associated with improved growth rates in the first two feeding phases of this trial. To validate this, it is necessary to analyze the metabolic profile of the intestinal epithelium. If this hypothesis is confirmed in future studies, it might be worth re-evaluating glutamine requirements for broilers fed with arginine above recommended levels.

Except for arginine, methionine, and glutamine, arginine supplementation did not significantly influence hepatic levels of other essential, conditionally essential, or non-essential AAs. The exposure to HS, however, modified the concentrations of many of them. HS decreased the levels of (iso)leucine, threonine, and valine in *P. major*, glycine and tyrosine in plasma and *P. major*, and aspartate and methionine in plasma, whereas increased the levels of glutamate, (iso)leucine, and threonine in liver, as well as those of phenylalanine in plasma and liver. These results broadly confirm that HS deeply alters protein metabolism in chickens, promoting the breakdown of skeletal muscle protein to supply the liver with AAs to be “burned” for energy (Zuo et al., 2015; Lu et al., 2018; Ma et al., 2021). Curiously, both arginine supplementation and HS reduced the concertation of another non-essential AA, that is alanine, in all tissues analyzed, apart from the liver when considering the effect of arginine supplementation. Similarly, HS decreased, in all tissues, the levels of N,N-Dimethylglycine, a betaine derivative involved in choline metabolism and synthesis of the antioxidant tripeptide glutathione by serving as a source of glycine (Simon, 1999; Kalmar et al., 2010). Our research group has found earlier a comparable reduction in N,N-Dimethylglycine in breast muscle of broilers reared under HS conditions (Zampiga et al., 2021). It has been reported that N, N-Dimethylglycine has free-radical scavenging properties (Hariganesh and Prathiba, 2010) and its use as a feed additive has been shown to produce positive effects on the health and performance of broilers (Kalmar et al., 2010; 2011; EFSA, 2011; Prola et al., 2013). These results, particularly the drop in N,N-Dimethylglycine levels caused by HS, and their potential implications for metabolism, growth, and responses to HS and oxidative stress would merit further in-depth investigations in broilers.

Oxidation-related molecules have a prominent role in the present discussion, which is not surprising considering the effects arginine and HS have been shown to have on oxidative stress (Akbarian et al., 2016; Wu et al., 2021). Arginine supplementation reduced the level of glutathione in the liver, as in our previous study (Brugaletta et al., 2023), and in *P. major*. Glutathione, composed of glutamate/glutamine, cysteine, and glycine, is very important for the antioxidant defense system, metabolism of nutrients, and regulation of cellular activities. Being the precursor of glutamate, arginine considerably influences the biosynthesis and levels of glutathione (Castro and Kim, 2020) that, like creatine, is mainly synthesized and provided by the liver (Wu et al., 2004). Considering this, increased hepatic glutathione levels would have been an expected consequence of arginine supplementation. The opposite outcome therefore needs further investigations. Energy-related molecules are also worth discussing. AMP was reduced in liver and *P. major* by arginine supplementation and was increased in *P. major* by HS. This nucleotide plays a pivotal role in many cellular metabolic processes, such as regulation of energy homeostasis by modulating the activity of the enzyme AMP-activated kinase (**AMPK**). Recognized as the master energy sensor for cells (Hardie, 2003; Hardie et al., 2006), AMPK senses energy levels by detecting modifications in the AMP to ATP ratio (Xiao et al., 2011; Chen et al., 2013). Under energy depletion (i.e., increased levels of AMP and decreased levels of ATP), AMP binds to the *γ* subunits of AMPK leading to the activation of this kinase that results in promotion of catabolic pathways and inhibition of anabolic pathways to generate ATP (Stein et al., 2000). Thus, the data from this study suggest that the liver and *P. major* of arginine-supplemented birds probably were in a good energy balance (low AMP levels), while *P. major* of heat-stressed birds suffered from energy depletion (high AMP levels). According to Baumgard and Rhoads (2013), negative energy balance and catabolic states are two of the most distinctive features of HS conditions. It is therefore intriguing that arginine supplementation may have been able to partially counterbalance the adverse effects of HS on energy homeostasis of broilers by increasing creatine levels and attenuating the increase in AMP levels, particularly in pectoral muscle. Non-etheless, the significant reduction in the levels of fumarate in all tissues and of succinate and UDP in plasma and liver supports the hypothesis that birds exposed to HS had a suboptimal energy balance. Succinate and fumarate are two consecutive intermediates in the citric acid cycle and, as such, their reduced availability can inhibit this central metabolic pathway, interfering with cellular bioenergetic processes. On the other hand, UDP is important in glycogenesis because it is combined with glucose to form UDP-glucose units that can be polymerized to glycogen chains. As UDP levels were reduced by HS, it can be assumed that glycogenesis, a chief anabolic pathway occurring in liver and muscles, was hampered in heat-stressed birds. In contrast, glycogenolysis, and gluconeogenesis may have been promoted to increase hepatic glucose production as reviewed by Rhoads et al. (2013), potentially resulting in the increased level of plasma glucose observed in heat-stressed birds.

Commenting on the results of cecal microbiota analysis, it is interesting to note that arginine supplementation significantly reduced observed diversity and the abundance of Actinobacteria, while increased the abundance of Bacilli unclassified. The change in observed diversity is similar to that obtained in our previous study by supplementing arginine to broilers reared under TN conditions (Brugaletta et al., 2023), but it is opposite to that of Singh et al. (2019) who found an increase in Shannon index in colonic samples taken from mice fed on a diet high in arginine. This inconsistency, however, may be due to the different animal species used and the origin of the intestinal content analyzed. The exposure to HS significantly increased alpha diversity indices (i.e., observed diversity, Shannon, and Inverse Simpson), which is consistent with the changes in ileal alpha diversity reported by Wang et al. (2018). HS was also found to significantly affect beta diversity, reduce the abundance of Bacteroidetes and increase that of Firmicutes, confirming the studies by Shi et al. (2019); Liu et al. (2020),; Goel et al. (2022). Moreover, the exposure to HS resulted in increased abundances of Enterobacteriaceae, Enterococcaceae, and Lachnospiraceae. The increase in Enterobacteriaceae, one of the most important members of the phylum Proteobacteria, caused by HS partly corroborates the work by Shi et al. (2019) who found that the abundance of cecal Proteobacteria increased in broilers exposed to HS. On the other hand, no results comparable to ours were found in the literature consulted regarding Enterococcaceae and Lachnospiraceae, so the effects of HS on these two cecal bacterial families in broilers are worth investigating further. Overall, the substantial variations in alpha and beta diversities and the results of taxonomic analysis indicate that the HS model applied here considerably affected the microbiota of broilers, broadly supporting previous research on this topic (Suzuki et al., 1983; Burkholder et al., 2008; Song et al., 2014; He et al., 2021; Liu et al., 2022). Interestingly, the factors group and room had a relevant interactive effect only on Lactobacillaceae, which were more abundant in birds fed the control diet and reared under HS conditions compared to the others. However, taking the cue from the study by Singh et al. (2019), further work is needed to elucidate how and to what extent arginine supplementation modulates the GI microbiota and its relationship with the broiler host under HS conditions.

In summary, this study shed light on some intricate metabolic and microbiota changes induced by arginine supplementation and cyclic HS in broilers, while also offering valuable starting points for future investigations that will undoubtedly help researchers better characterize the response of broilers to these two factors and their interaction.

## Data Availability

The datasets presented in this study can be found in online repositories. The names of the repository/repositories and accession number(s) can be found below: Metagenome sequence data—https://www.ncbi.nlm.nih.gov/, Bioproject PRJNA928248.

## Abbreviations

^1^H-NMR, proton nuclear magnetic resonance; AA, amino acid; AMPK, AMP-activated kinase; ARG, arginine-supplemented group; BW, body weight; CON, control group; DFI, daily feed intake; DWG, daily weight gain; FCR, feed conversion ratio; FI, feed intake; GI, gastrointestinal; HS, heat stress; RH, relative humidity; TN, thermoneutral.
