# Supplementary material for: Metabolic and microbiota response to arginine supplementation and cyclic heat stress in broiler chickens
Source: Front Physiol. 2023 Mar 31;14:1155324. doi: 10.3389/fphys.2023.1155324 (PMC10102354; doi:10.3389/fphys.2023.1155324)
Supplement: Supplementary file 1 [file Table1.DOCX]

Supplementary Material

Metabolic and Microbiota Response to Arginine Supplementation and Cyclic Heat Stress in Broiler Chickens

**Giorgio Brugaletta, Luca Laghi, Marco Zampiga, Chiara Oliveri, Valentina Indio, Raffaela Piscitelli, Stefano Pignata, Massimiliano Petracci, Alessandra De Cesare, Federico Sirri** Correspondence:** Federico Sirri: [federico.sirri@unibo.it](mailto:federico.sirri@unibo.it)

Please see below.

Supplementary Table 1. Metabolites showing different concentrations (mmol/L) in the plasma of CON and ARG birds at D44

| **Metabolite** | **Group** | | **Room** | | **Group × Room** | | | **SE** | | ***P*-value** | | |  |
| --- | --- | --- | --- | --- | --- | --- | --- | --- | --- | --- | --- | --- | --- |
|  | **CON** | **ARG** | **TN** | **HS** | **CON-TN** | **ARG-TN** | **CON-HS** | **ARG-HS** |  | **Group** | **Room** | **Group × Room** | |
| 2,3-Butanediol | 4.25E-02 | 3.96E-02 | 3.37E-02 | 4.91E-02 | 3.47E-02 | 3.27E-02 | 5.11E-02 | 4.70E-02 | 2.16E-02 | 0.591 | **0.021** | 0.991 | |
| 3-Hydroxyisobutyrate | 1.56E-02 | 1.60E-02 | 1.60E-02 | 1.56E-02 | 1.68E-02 | 1.52E-02 | 1.42E-02 | 1.70E-02 | 4.32E-03 | 0.729 | 0.745 | 0.092 | |
| 3-Methyl-2-oxovalerate | 8.23E-03 | 7.97E-03 | 6.89E-03 | 9.41E-03 | 7.45E-03 | 6.32E-03 | 9.07E-03 | 9.76E-03 | 3.93E-03 | 0.863 | 0.087 | 0.227 | |
| Acetone | 2.27E-02 | 2.13E-02 | 1.99E-02 | 2.43E-02 | 2.05E-02 | 1.94E-02 | 2.51E-02 | 2.35E-02 | 7.73E-03 | 0.559 | 0.098 | 0.983 | |
| Alanine | 1.03E+00 | 8.95E-01 | 1.08E+00 | 8.36E-01 | 1.18E+00 | 9.78E-01 | 8.68E-01 | 8.05E-01 | 1.94E-01 | **0.022** | **0.000** | 0.227 | |
| Arabinose | 7.52E-02 | 7.41E-02 | 7.10E-02 | 7.86E-02 | 7.21E-02 | 6.99E-02 | 7.86E-02 | 7.86E-02 | 1.28E-02 | 0.765 | 0.051 | 0.769 | |
| Arginine | 2.02E-01 | 3.31E-01 | 2.86E-01 | 2.46E-01 | 2.24E-01 | 3.48E-01 | 1.79E-01 | 3.13E-01 | 1.03E-01 | **0.000** | 0.195 | 0.862 | |
| Aspartate | 1.15E-01 | 1.09E-01 | 1.26E-01 | 9.73E-02 | 1.33E-01 | 1.19E-01 | 9.54E-02 | 9.93E-02 | 4.13E-02 | 0.830 | **0.044** | 0.607 | |
| Betaine | 8.55E-01 | 8.69E-01 | 9.07E-01 | 8.13E-01 | 9.07E-01 | 9.08E-01 | 7.99E-01 | 8.27E-01 | 1.46E-01 | 0.599 | **0.048** | 0.968 | |
| Carnosine | 5.41E-02 | 4.72E-02 | 5.90E-02 | 4.15E-02 | 6.71E-02 | 5.08E-02 | 3.98E-02 | 4.33E-02 | 2.00E-02 | 0.378 | **0.008** | 0.095 | |
| Creatine | 8.48E-02 | 1.10E-01 | 1.06E-01 | 8.77E-02 | 1.02E-01 | 1.10E-01 | 6.61E-02 | 1.09E-01 | 6.11E-02 | **0.032** | 0.074 | 0.395 | |
| Ethanol | 7.33E-02 | 5.08E-02 | 3.43E-02 | 9.24E-02 | 3.94E-02 | 2.92E-02 | 1.10E-01 | 7.43E-02 | 6.26E-02 | 0.180 | **0.000** | 0.754 | |
| Formate | 9.53E-02 | 1.02E-01 | 7.12E-02 | 1.29E-01 | 6.31E-02 | 7.92E-02 | 1.30E-01 | 1.27E-01 | 4.14E-02 | 0.130 | **0.000** | 0.068 | |
| Fumarate | 8.65E-03 | 8.08E-03 | 9.23E-03 | 7.43E-03 | 9.36E-03 | 9.09E-03 | 7.87E-03 | 6.98E-03 | 2.77E-03 | 0.490 | **0.033** | 0.707 | |
| Glucose | 1.42E+01 | 1.44E+01 | 1.34E+01 | 1.53E+01 | 1.32E+01 | 1.36E+01 | 1.54E+01 | 1.52E+01 | 1.62E+00 | 0.688 | **0.000** | 0.528 | |
| Glutamine | 1.46E+00 | 1.27E+00 | 1.42E+00 | 1.31E+00 | 1.48E+00 | 1.36E+00 | 1.43E+00 | 1.18E+00 | 2.33E-01 | **0.010** | 0.102 | 0.385 | |
| Glycerol | 1.40E-01 | 1.36E-01 | 1.62E-01 | 1.11E-01 | 1.72E-01 | 1.53E-01 | 1.05E-01 | 1.17E-01 | 5.50E-02 | 0.903 | **0.001** | 0.481 | |
| Glycine | 7.13E-01 | 6.77E-01 | 7.43E-01 | 6.42E-01 | 7.80E-01 | 7.07E-01 | 6.40E-01 | 6.45E-01 | 1.13E-01 | 0.290 | **0.004** | 0.248 | |
| Mannose | 5.67E-02 | 5.61E-02 | 4.29E-02 | 7.12E-02 | 4.42E-02 | 4.15E-02 | 7.03E-02 | 7.21E-02 | 2.17E-02 | 0.552 | **0.000** | 0.306 | |
| Methionine | 1.34E-01 | 1.37E-01 | 1.54E-01 | 1.16E-01 | 1.52E-01 | 1.56E-01 | 1.15E-01 | 1.17E-01 | 3.63E-02 | 0.790 | **0.001** | 0.913 | |
| N,N-Dimethylglycine | 1.19E-01 | 1.12E-01 | 1.20E-01 | 1.10E-01 | 1.23E-01 | 1.18E-01 | 1.14E-01 | 1.05E-01 | 1.80E-02 | 0.190 | 0.052 | 0.755 | |
| Phenylalanine | 1.32E-01 | 1.34E-01 | 1.27E-01 | 1.39E-01 | 1.24E-01 | 1.29E-01 | 1.40E-01 | 1.39E-01 | 1.72E-02 | 0.678 | **0.019** | 0.553 | |
| Pyroglutamate | 8.72E-02 | 8.19E-02 | 7.94E-02 | 9.01E-02 | 8.04E-02 | 7.85E-02 | 9.46E-02 | 8.56E-02 | 2.38E-02 | 0.602 | **0.009** | 0.233 | |
| Sarcosine | 2.92E-02 | 2.66E-02 | 3.00E-02 | 2.56E-02 | 3.36E-02 a | 2.64E-02 b | 2.44E-02 b | 2.67E-02 b | 5.05E-03 | 0.085 | **0.004** | **0.003** | |
| Serine | 8.16E-01 | 8.01E-01 | 7.55E-01 | 8.66E-01 | 7.41E-01 | 7.70E-01 | 8.97E-01 | 8.36E-01 | 1.35E-01 | 0.715 | **0.008** | 0.262 | |
| Succinate | 5.73E-02 | 5.46E-02 | 6.20E-02 | 4.93E-02 | 6.39E-02 | 6.00E-02 | 5.00E-02 | 4.87E-02 | 1.60E-02 | 0.749 | **0.013** | 0.740 | |
| Threonine | 8.50E-01 | 7.70E-01 | 7.75E-01 | 8.48E-01 | 8.46E-01 | 7.04E-01 | 8.54E-01 | 8.43E-01 | 1.51E-01 | 0.081 | 0.108 | 0.148 | |
| Tyrosine | 1.65E-01 | 1.56E-01 | 1.72E-01 | 1.49E-01 | 1.68E-01 | 1.75E-01 | 1.62E-01 | 1.36E-01 | 4.47E-02 | 0.514 | 0.090 | 0.214 | |

*Note:* *n* = 12 birds/group/room. *P*-values less than 0.05 are in bold. Means that fall under the interaction between group and room and show distinct letters are significantly different (*P* < 0.05).

Supplementary Table 2. Metabolites showing different concentrations (mmol/L) in the liver of CON and ARG birds at D44

| **Metabolite** | **Group** | | **Room** | | **Group × Room** | | | | **SE** | ***P*-value** | | |
| --- | --- | --- | --- | --- | --- | --- | --- | --- | --- | --- | --- | --- |
|  | **CON** | **ARG** | **TN** | **HS** | **CON-TN** | **ARG-TN** | **CON-HS** | **ARG-HS** |  | **Group** | **Room** | **Group × Room** |
| 1-Methylhistidine | 5.26E-04 | 5.40E-04 | 4.77E-04 | 5.96E-04 | 4.73E-04 | 4.81E-04 | 5.91E-04 | 6.00E-04 | 1.30E-04 | 0.717 | **0.004** | 1.000 |
| 1,3-Dihydroxyacetone | 7.65E-05 | 5.96E-05 | 6.52E-05 | 7.04E-05 | 7.66E-05 | 5.38E-05 | 7.64E-05 | 6.54E-05 | 2.30E-05 | **0.043** | 0.202 | 0.366 |
| 3-Hydroxybutyrate | 9.17E-04 | 7.28E-04 | 9.42E-04 | 6.84E-04 | 1.01E-03 | 8.74E-04 | 8.07E-04 | 5.81E-04 | 2.87E-04 | **0.011** | **0.006** | 0.403 |
| 4-Aminobutyrate | 4.87E-04 | 5.76E-04 | 5.37E-04 | 5.29E-04 | 4.90E-04 | 5.85E-04 | 4.84E-04 | 5.67E-04 | 1.48E-04 | 0.056 | 0.753 | 0.994 |
| Adenine | 6.78E-04 | 5.11E-04 | 5.70E-04 | 6.14E-04 | 6.34E-04 | 5.05E-04 | 7.30E-04 | 5.17E-04 | 2.53E-04 | 0.052 | 0.435 | 0.506 |
| Alanine | 5.14E-03 | 4.60E-03 | 5.26E-03 | 4.42E-03 | 5.64E-03 | 4.88E-03 | 4.55E-03 | 4.32E-03 | 1.12E-03 | 0.106 | **0.018** | 0.427 |
| AMP | 2.86E-03 | 2.32E-03 | 2.68E-03 | 2.46E-03 | 2.98E-03 | 2.39E-03 | 2.71E-03 | 2.26E-03 | 4.84E-04 | **0.001** | 0.175 | 0.619 |
| Arginine | 9.16E-04 | 1.29E-03 | 1.07E-03 | 1.16E-03 | 9.25E-04 | 1.21E-03 | 9.05E-04 | 1.37E-03 | 3.25E-04 | **0.000** | 0.467 | 0.377 |
| Creatine | 7.81E-04 | 1.05E-03 | 9.99E-04 | 8.31E-04 | 7.64E-04 | 1.23E-03 | 8.02E-04 | 8.55E-04 | 6.83E-04 | **0.019** | **0.033** | 0.544 |
| Dimethylamine | 2.00E-04 | 2.37E-04 | 2.29E-04 | 2.09E-04 | 2.15E-04 | 2.43E-04 | 1.83E-04 | 2.30E-04 | 5.10E-05 | **0.019** | 0.155 | 0.538 |
| Ethanol | 1.20E-02 | 1.99E-02 | 1.48E-02 | 1.75E-02 | 1.09E-02 | 1.87E-02 | 1.32E-02 | 2.10E-02 | 1.23E-02 | **0.029** | 0.854 | 0.952 |
| Ethanolamine | 7.33E-04 | 8.89E-04 | 7.82E-04 | 8.49E-04 | 5.71E-04 | 9.93E-04 | 9.27E-04 | 7.85E-04 | 5.09E-04 | 0.148 | 0.466 | 0.058 |
| Fumarate | 1.08E-03 | 1.09E-03 | 1.16E-03 | 9.94E-04 | 1.13E-03 | 1.20E-03 | 1.02E-03 | 9.75E-04 | 2.19E-04 | 0.967 | **0.011** | 0.380 |
| Glutamate | 6.83E-03 | 6.51E-03 | 6.11E-03 | 7.27E-03 | 6.14E-03 | 6.08E-03 | 7.66E-03 | 6.94E-03 | 1.31E-03 | 0.412 | **0.004** | 0.408 |
| Glutamine | 7.16E-03 | 5.84E-03 | 5.31E-03 | 7.74E-03 | 5.70E-03 | 4.91E-03 | 8.90E-03 | 6.77E-03 | 2.09E-03 | 0.063 | **0.000** | 0.363 |
| Glutathione | 2.16E-03 | 1.76E-03 | 2.20E-03 | 1.68E-03 | 2.45E-03 | 1.94E-03 | 1.81E-03 | 1.57E-03 | 4.88E-04 | **0.010** | **0.001** | 0.481 |
| Hypoxanthine | 3.09E-04 | 2.36E-04 | 2.66E-04 | 2.76E-04 | 2.84E-04 | 2.48E-04 | 3.38E-04 | 2.25E-04 | 1.12E-04 | 0.078 | 0.631 | 0.221 |
| Isoleucine | 3.89E-04 | 4.27E-04 | 3.73E-04 | 4.49E-04 | 3.72E-04 | 3.74E-04 | 4.10E-04 | 4.81E-04 | 1.33E-04 | 0.335 | 0.065 | 0.384 |
| Isopropanol | 9.71E-05 | 1.65E-04 | 1.17E-04 | 1.50E-04 | 8.44E-05 | 1.49E-04 | 1.12E-04 | 1.81E-04 | 9.21E-05 | **0.007** | 0.490 | 0.945 |
| Leucine | 9.79E-04 | 1.13E-03 | 9.58E-04 | 1.17E-03 | 9.40E-04 | 9.75E-04 | 1.02E-03 | 1.28E-03 | 3.67E-04 | 0.172 | 0.070 | 0.309 |
| Methionine | 3.86E-04 | 4.60E-04 | 4.06E-04 | 4.45E-04 | 3.86E-04 | 4.26E-04 | 3.86E-04 | 4.95E-04 | 1.42E-04 | 0.083 | 0.392 | 0.410 |
| myo-Inositol | 3.57E-02 | 3.21E-02 | 3.56E-02 | 3.19E-02 | 3.75E-02 | 3.37E-02 | 3.35E-02 | 3.05E-02 | 6.15E-03 | 0.056 | 0.053 | 0.815 |
| N,N-Dimethylglycine | 1.27E-04 | 1.12E-04 | 1.33E-04 | 1.05E-04 | 1.37E-04 | 1.29E-04 | 1.16E-04 | 9.57E-05 | 3.11E-05 | 0.055 | **0.003** | 0.323 |
| NAD+ | 6.10E-04 | 4.46E-04 | 5.00E-04 | 5.51E-04 | 5.61E-04 | 4.39E-04 | 6.69E-04 | 4.53E-04 | 2.18E-04 | **0.014** | 0.364 | 0.466 |
| Pantothenate | 1.05E-04 | 8.52E-05 | 1.02E-04 | 8.63E-05 | 1.09E-04 | 9.48E-05 | 9.91E-05 | 7.57E-05 | 2.34E-05 | **0.007** | **0.037** | 0.537 |
| Phenylalanine | 5.78E-04 | 6.72E-04 | 5.61E-04 | 7.00E-04 | 5.46E-04 | 5.75E-04 | 6.17E-04 | 7.69E-04 | 2.20E-04 | 0.119 | **0.041** | 0.422 |
| Propylene glycol | 9.69E-05 | 8.23E-05 | 8.60E-05 | 9.28E-05 | 8.30E-05 b | 8.91E-05 ab | 1.14E-04 a | 7.55E-05 b | 2.17E-05 | 0.053 | 0.398 | **0.001** |
| Pyroglutamate | 3.14E-04 | 3.06E-04 | 2.50E-04 | 3.75E-04 | 2.40E-04 | 2.60E-04 | 4.02E-04 | 3.52E-04 | 1.22E-04 | 0.834 | **0.001** | 0.328 |
| Sarcosine | 1.14E-04 | 9.42E-05 | 1.22E-04 | 8.38E-05 | 1.42E-04 | 1.02E-04 | 8.12E-05 | 8.59E-05 | 3.79E-05 | 0.099 | **0.001** | 0.095 |
| Succinate | 3.01E-03 | 2.92E-03 | 3.16E-03 | 2.75E-03 | 3.16E-03 | 3.16E-03 | 2.83E-03 | 2.69E-03 | 5.98E-04 | 0.626 | **0.029** | 0.687 |
| Threonine | 1.75E-03 | 1.84E-03 | 1.64E-03 | 1.96E-03 | 1.68E-03 | 1.61E-03 | 1.84E-03 | 2.07E-03 | 5.41E-04 | 0.591 | 0.054 | 0.361 |
| UDP | 2.25E-04 | 1.96E-04 | 2.35E-04 | 1.82E-04 | 2.51E-04 | 2.19E-04 | 1.94E-04 | 1.73E-04 | 3.17E-05 | **0.003** | **0.000** | 0.582 |
| Uracil | 1.31E-04 | 1.67E-04 | 1.26E-04 | 1.77E-04 | 1.15E-04 | 1.36E-04 | 1.51E-04 | 1.98E-04 | 5.81E-05 | **0.026** | **0.005** | 0.830 |
| Uridine | 4.93E-04 | 5.00E-04 | 4.62E-04 | 5.35E-04 | 4.74E-04 | 4.51E-04 | 5.16E-04 | 5.50E-04 | 1.05E-04 | 0.821 | **0.025** | 0.373 |

*Note:* *n* = 12 birds/group/room. *P*-values less than 0.05 are in bold. Means that fall under the interaction between group and room and show distinct letters are significantly different (*P* < 0.05).

Supplementary Table 3. Metabolites showing different concentrations (mmol/L) in the *Pectoralis major* of CON and ARG birds at D44

| **Metabolite** | **Group** | | **Room** | | **Group × Room** | | | | | | | **SE** | | | ***P*-value** | | |  |  |
| --- | --- | --- | --- | --- | --- | --- | --- | --- | --- | --- | --- | --- | --- | --- | --- | --- | --- | --- | --- |
|  | **CON** | **ARG** | **TN** | **HS** | **CON-TN** | | **ARG-TN** | | **CON-HS** | | **ARG-HS** |  |  |  | **Group** | | **Room** | **Group × Room** | |
| 2-Aminobutyrate | 9.22E-05 | 9.31E-05 | 1.13E-04 | 7.26E-05 | 1.11E-04 | 1.14E-04 | | 7.30E-05 | 7.21E-05 | 5.45E-05 | 0.600 | **0.003** | | 0.744 | | |  |  |  |
| 2-Oxoglutarate | 7.58E-04 | 5.69E-04 | 4.52E-04 | 8.75E-04 | 5.22E-04 | 3.82E-04 | | 9.94E-04 | 7.57E-04 | 4.56E-04 | 0.172 | **0.002** | | 0.947 | | |  |  |  |
| 2,3-Butanediol | 5.79E-05 | 6.28E-05 | 5.50E-05 | 6.57E-05 | 4.87E-05 | 6.13E-05 | | 6.71E-05 | 6.43E-05 | 1.95E-05 | 0.388 | 0.065 | | 0.178 | | |  |  |  |
| 3-Hydroxyisovalerate | 1.74E-05 | 1.96E-05 | 1.76E-05 | 1.94E-05 | 1.63E-05 | 1.90E-05 | | 1.85E-05 | 2.03E-05 | 3.21E-06 | **0.022** | 0.063 | | 0.621 | | |  |  |  |
| 4-Aminobutyrate | 1.19E-04 | 8.97E-05 | 9.48E-05 | 1.14E-04 | 1.09E-04 | 8.07E-05 | | 1.29E-04 | 9.88E-05 | 3.93E-05 | **0.013** | 0.098 | | 0.927 | | |  |  |  |
| 4-Hydroxyphenylacetate | 3.09E-04 | 1.85E-04 | 2.37E-04 | 2.57E-04 | 2.88E-04 | 1.86E-04 | | 3.29E-04 | 1.85E-04 | 1.38E-04 | **0.004** | 0.779 | | 0.844 | | |  |  |  |
| Acetate | 2.17E-04 | 3.15E-04 | 2.67E-04 | 2.66E-04 | 2.15E-04 | 3.18E-04 | | 2.19E-04 | 3.12E-04 | 6.78E-05 | **0.000** | 0.961 | | 0.803 | | |  |  |  |
| Acetone | 1.85E-05 | 1.59E-05 | 1.61E-05 | 1.83E-05 | 1.71E-05 | 1.52E-05 | | 2.00E-05 | 1.66E-05 | 5.09E-06 | 0.083 | 0.152 | | 0.619 | | |  |  |  |
| Alanine | 3.03E-03 | 2.68E-03 | 3.27E-03 | 2.44E-03 | 3.58E-03 | 2.96E-03 | | 2.47E-03 | 2.40E-03 | 6.76E-04 | 0.083 | **0.000** | | 0.161 | | |  |  |  |
| AMP | 2.96E-04 | 2.36E-04 | 2.35E-04 | 2.97E-04 | 2.50E-04 | 2.21E-04 | | 3.42E-04 | 2.52E-04 | 9.21E-05 | **0.030** | **0.025** | | 0.261 | | |  |  |  |
| Anserine | 2.51E-02 | 2.70E-02 | 2.30E-02 | 2.91E-02 | 2.33E-02 | 2.27E-02 | | 2.69E-02 | 3.12E-02 | 5.81E-03 | 0.454 | **0.001** | | 0.142 | | |  |  |  |
| Arginine | 1.92E-04 | 2.17E-04 | 2.08E-04 | 2.01E-04 | 1.93E-04 | 2.24E-04 | | 1.91E-04 | 2.11E-04 | 7.72E-05 | 0.068 | 0.423 | | 0.919 | | |  |  |  |
| Asparagine | 6.93E-04 | 5.48E-04 | 7.22E-04 | 5.20E-04 | 8.14E-04 | 6.29E-04 | | 5.72E-04 | 4.67E-04 | 2.49E-04 | 0.050 | **0.007** | | 0.584 | | |  |  |  |
| Beta-Alanine | 5.41E-03 | 4.45E-03 | 3.72E-03 | 6.14E-03 | 3.71E-03 | 3.72E-03 | | 7.10E-03 | 5.17E-03 | 2.25E-03 | 0.147 | **0.001** | | 0.141 | | |  |  |  |
| Carnosine | 4.19E-04 | 4.00E-04 | 3.22E-04 | 4.96E-04 | 3.36E-04 | 3.08E-04 | | 5.01E-04 | 4.92E-04 | 1.37E-04 | 0.641 | **0.000** | | 0.815 | | |  |  |  |
| Choline | 2.25E-04 | 2.21E-04 | 1.92E-04 | 2.54E-04 | 1.95E-04 | 1.89E-04 | | 2.55E-04 | 2.54E-04 | 8.43E-05 | 0.890 | **0.014** | | 0.918 | | |  |  |  |
| Creatine | 4.93E-02 | 5.89E-02 | 4.87E-02 | 5.95E-02 | 4.47E-02 | 5.28E-02 | | 5.40E-02 | 6.50E-02 | 9.83E-03 | **0.002** | **0.000** | | 0.621 | | |  |  |  |
| Dimethyl sulfone | 1.72E-04 | 1.96E-04 | 1.67E-04 | 2.01E-04 | 1.54E-04 | 1.81E-04 | | 1.91E-04 | 2.10E-04 | 3.20E-05 | **0.016** | **0.001** | | 0.693 | | |  |  |  |
| Ethanolamine | 9.43E-04 | 9.24E-04 | 1.08E-03 | 7.85E-04 | 1.12E-03 | 1.05E-03 | | 7.71E-04 | 8.00E-04 | 5.10E-04 | 0.896 | 0.050 | | 0.746 | | |  |  |  |
| Formate | 1.50E-04 | 1.73E-04 | 1.62E-04 | 1.61E-04 | 1.47E-04 | 1.76E-04 | | 1.52E-04 | 1.70E-04 | 4.29E-05 | 0.068 | 0.957 | | 0.691 | | |  |  |  |
| Fumarate | 7.07E-05 | 6.89E-05 | 7.71E-05 | 6.24E-05 | 7.81E-05 | 7.61E-05 | | 6.33E-05 | 6.16E-05 | 2.39E-05 | 0.792 | **0.039** | | 0.984 | | |  |  |  |
| Glucose-1-phosphate | 4.53E-04 | 2.51E-03 | 2.51E-03 | 4.58E-04 | 4.86E-04 | 4.53E-03 | | 4.19E-04 | 4.97E-04 | 6.91E-03 | 0.070 | 0.125 | | 0.998 | | |  |  |  |
| Glutamine | 3.20E-03 | 2.78E-03 | 3.43E-03 | 2.55E-03 | 3.68E-03 | 3.19E-03 | | 2.72E-03 | 2.38E-03 | 8.59E-04 | 0.099 | **0.001** | | 0.769 | | |  |  |  |
| Glutathione | 5.52E-04 | 3.84E-04 | 4.67E-04 | 4.69E-04 | 5.95E-04 | 3.40E-04 | | 5.09E-04 | 4.29E-04 | 1.97E-04 | **0.002** | 0.568 | | 0.216 | | |  |  |  |
| Glycerol | 5.31E-04 | 7.96E-04 | 6.90E-04 | 6.37E-04 | 5.19E-04 | 8.61E-04 | | 5.42E-04 | 7.31E-04 | 3.84E-04 | **0.016** | 0.783 | | 0.351 | | |  |  |  |
| Glycine | 4.56E-03 | 4.31E-03 | 5.48E-03 | 3.38E-03 | 5.69E-03 | 5.28E-03 | | 3.43E-03 | 3.34E-03 | 1.73E-03 | 0.859 | **0.000** | | 0.865 | | |  |  |  |
| Isoleucine | 8.13E-05 | 8.45E-05 | 9.94E-05 | 6.64E-05 | 1.03E-04 | 9.54E-05 | | 5.93E-05 | 7.36E-05 | 2.97E-05 | 0.359 | **0.000** | | 0.059 | | |  |  |  |
| Lactate | 9.50E-02 | 1.08E-01 | 9.57E-02 | 1.08E-01 | 9.01E-02 | 1.01E-01 | | 9.99E-02 | 1.15E-01 | 2.09E-02 | **0.031** | 0.067 | | 0.802 | | |  |  |  |
| Leucine | 1.41E-04 | 1.52E-04 | 1.71E-04 | 1.22E-04 | 1.67E-04 | 1.75E-04 | | 1.15E-04 | 1.28E-04 | 5.13E-05 | 0.531 | **0.001** | | 0.665 | | |  |  |  |
| Malonate | 4.83E-04 | 6.09E-04 | 5.07E-04 | 5.85E-04 | 4.58E-04 | 5.55E-04 | | 5.07E-04 | 6.63E-04 | 1.33E-04 | **0.002** | **0.047** | | 0.441 | | |  |  |  |
| Methionine sulfoxide | 6.26E-05 | 1.42E-04 | 7.97E-05 | 1.25E-04 | 5.77E-05 | 1.02E-04 | | 6.75E-05 | 1.83E-04 | 1.08E-04 | **0.010** | 0.208 | | 0.701 | | |  |  |  |
| N,N-Dimethylglycine | 1.05E-03 | 9.96E-04 | 1.12E-03 | 9.29E-04 | 1.10E-03 | 1.14E-03 | | 1.00E-03 | 8.54E-04 | 2.77E-04 | 0.491 | **0.021** | | 0.244 | | |  |  |  |
| N2-Acetyllysine | 5.39E-04 | 8.83E-04 | 5.40E-04 | 8.82E-04 | 4.02E-04 | 6.78E-04 | | 6.77E-04 | 1.09E-03 | 5.89E-04 | 0.068 | 0.056 | | 0.440 | | |  |  |  |
| NAD | 8.47E-04 | 8.12E-04 | 7.35E-04 | 9.24E-04 | 7.46E-04 | 7.25E-04 | | 9.48E-04 | 9.00E-04 | 2.04E-04 | 0.653 | **0.002** | | 0.867 | | |  |  |  |
| Niacinamide | 1.08E-04 | 2.46E-04 | 2.02E-04 | 1.52E-04 | 1.12E-04 | 2.92E-04 | | 1.04E-04 | 2.00E-04 | 2.78E-04 | 0.094 | 0.536 | | 0.602 | | |  |  |  |
| Proline | 7.75E-04 | 1.31E-03 | 7.89E-04 | 1.29E-03 | 5.07E-04 | 1.07E-03 | | 1.04E-03 | 1.55E-03 | 1.15E-03 | **0.043** | **0.036** | | 0.563 | | |  |  |  |
| Serine | 1.38E-03 | 1.27E-03 | 1.40E-03 | 1.25E-03 | 1.44E-03 | 1.36E-03 | | 1.32E-03 | 1.19E-03 | 4.31E-04 | 0.089 | 0.288 | | 0.713 | | |  |  |  |
| Threonine | 8.03E-04 | 7.08E-04 | 8.56E-04 | 6.54E-04 | 9.81E-04 | 7.31E-04 | | 6.24E-04 | 6.84E-04 | 2.88E-04 | 0.260 | **0.020** | | 0.069 | | |  |  |  |
| Tyrosine | 4.19E-04 | 3.94E-04 | 4.65E-04 | 3.47E-04 | 4.76E-04 | 4.54E-04 | | 3.61E-04 | 3.33E-04 | 1.23E-04 | 0.489 | **0.002** | | 0.932 | | |  |  |  |
| UDP | 1.57E-04 | 6.43E-04 | 6.62E-04 | 1.38E-04 | 1.85E-04 | 1.14E-03 | | 1.30E-04 | 1.46E-04 | 1.60E-03 | 0.084 | **0.003** | | 0.298 | | |  |  |  |
| Valine | 1.86E-04 | 2.02E-04 | 2.26E-04 | 1.61E-04 | 2.19E-04 | 2.33E-04 | | 1.52E-04 | 1.70E-04 | 5.40E-05 | 0.311 | **0.000** | | 0.909 | | |  |  |  |

*Note:* *n* = 12 birds/group/room. *P*-values less than 0.05 are in bold.

**Supplementary Table 4.**

|  | | | Mean relative abundance % | | | | | | | | | | | | | | | Two-way ANOVA *P*-values | | | | | | | Tukey’s HSD post-hoc test *P*-values | | | | | | | | | | | |  |
| --- | --- | --- | --- | --- | --- | --- | --- | --- | --- | --- | --- | --- | --- | --- | --- | --- | --- | --- | --- | --- | --- | --- | --- | --- | --- | --- | --- | --- | --- | --- | --- | --- | --- | --- | --- | --- | --- |
| **Phylum** | CON | | | | ARG | | HS | | TN | | | ARG_HS | | CON_HS | | ARG_TN | CON_TN | | Room | | Group | | Room **×** Group | | | ARG_HS vs ARG_TN | | ARG_HS vs CON_HS | | CON_TN vs ARG_HS | | CON_HS vs ARG_TN | | CON_TN vs ARG_TN | | CON_TN vs CON_HS |  |
| Actinobacteria | 1.52 | | | | 0.78 | | 1.14 | | 1.15 | | 0.62 | | | 1.67 | | 0.93 | 1.37 | | 0.985 | | 0.008 | | 0.254 | | | 0.840 | | 0.037 | | 0.207 | | 0.217 | | 0.655 | | 0.852 |  |
| Bacteria_unclassified | 0.04 | | | | 0.06 | | 0.06 | | 0.04 | | 0.05 | | | 0.07 | | 0.06 | 0.02 | | 0.336 | | 0.654 | | 0.187 | | | 0.994 | | 0.923 | | 0.746 | | 0.983 | | 0.589 | | 0.370 |  |
| Bacteroidetes | 6.82 | | | | 7.63 | | 6.17 | | 8.28 | | 6.75 | | | 5.59 | | 8.52 | 8.05 | | 0.081 | | 0.495 | | 0.770 | | | 0.719 | | 0.899 | | 0.865 | | 0.312 | | 0.992 | | 0.464 |  |
| Candidatus_Melainabacteria | 0.02 | | | | 0.07 | | 0.03 | | 0.06 | | 0.04 | | | 0.02 | | 0.09 | 0.02 | | 0.322 | | 0.137 | | 0.362 | | | 0.530 | | 0.975 | | 0.983 | | 0.296 | | 0.324 | | 1.000 |  |
| Cyanobacteria | 0.37 | | | | 0.49 | | 0.32 | | 0.54 | | 0.42 | | | 0.22 | | 0.56 | 0.52 | | 0.129 | | 0.393 | | 0.574 | | | 0.899 | | 0.744 | | 0.962 | | 0.333 | | 0.997 | | 0.449 |  |
| Firmicutes | 89.28 | | | | 88.04 | | 90.57 | | 86.75 | | 90.67 | | | 90.46 | | 85.40 | 88.10 | | 0.027 | | 0.461 | | 0.390 | | | 0.131 | | 1.000 | | 0.698 | | 0.156 | | 0.667 | | 0.750 |  |
| Proteobacteria | 1.78 | | | | 1.49 | | 1.68 | | 1.60 | | 1.42 | | | 1.93 | | 1.57 | 1.63 | | 0.730 | | 0.216 | | 0.323 | | | 0.967 | | 0.392 | | 0.918 | | 0.672 | | 0.998 | | 0.777 |  |
| Verrucomicrobia | 0.17 | | | | 1.45 | | 0.03 | | 1.59 | | 0.03 | | | 0.03 | | 2.87 | 0.31 | | 0.147 | | 0.232 | | 0.230 | | | 0.241 | | 1.000 | | 0.998 | | 0.243 | | 0.327 | | 0.998 |  |
|  | |  | |  | |  | |  | |  | | |  | |  | |  | |  |  | |  | |  | | |  | |  | |  | |  | |  | | |
| **Family** | | | | | | | | | | | | | | | | | | | | | | | | | | | | | | | | | | | | |  |
| Akkermansiaceae | 0.2 | | | | 1.4 | | 0.0 | | 1.6 | | 0.0 | | | 0.0 | | 2.9 | 0.3 | | 0.147 | | 0.232 | | 0.230 | | | 0.241 | | 1.000 | | 0.998 | | 0.243 | | 0.327 | | 0.998 |  |
| Bacilli_unclassified | 0.2 | | | | 0.7 | | 0.5 | | 0.4 | | 0.8 | | | 0.3 | | 0.6 | 0.2 | | 0.275 | | 0.001 | | 0.617 | | | 0.668 | | 0.032 | | 0.010 | | 0.325 | | 0.154 | | 0.974 |  |
| Bacteria_unclassified | 0.0 | | | | 0.1 | | 0.1 | | 0.0 | | 0.1 | | | 0.1 | | 0.1 | 0.0 | | 0.336 | | 0.654 | | 0.187 | | | 0.994 | | 0.923 | | 0.746 | | 0.983 | | 0.589 | | 0.370 |  |
| Bacteroidaceae | 0.8 | | | | 0.8 | | 0.7 | | 0.8 | | 0.5 | | | 1.0 | | 1.1 | 0.6 | | 0.716 | | 0.979 | | 0.072 | | | 0.409 | | 0.553 | | 0.992 | | 0.995 | | 0.576 | | 0.724 |  |
| Bifidobacteriaceae | 0.0 | | | | 0.0 | | 0.0 | | 0.0 | | 0.0 | | | 0.0 | | 0.0 | 0.0 | | 0.396 | | 0.418 | | 0.258 | | | 0.497 | | 0.996 | | 1.000 | | 0.640 | | 0.514 | | 0.997 |  |
| Candidatus_Gastranaerophilales_unclassified | 0.0 | | | | 0.1 | | 0.0 | | 0.1 | | 0.0 | | | 0.0 | | 0.1 | 0.0 | | 0.322 | | 0.137 | | 0.362 | | | 0.530 | | 0.975 | | 0.983 | | 0.296 | | 0.324 | | 1.000 |  |
| Christensenellaceae | 0.0 | | | | 0.0 | | 0.0 | | 0.0 | | 0.0 | | | 0.0 | | 0.0 | 0.0 | | 0.283 | | 0.963 | | 0.750 | | | 0.753 | | 0.994 | | 0.853 | | 0.882 | | 0.997 | | 0.948 |  |
| Clostridia_unclassified | 17.9 | | | | 17.5 | | 18.3 | | 17.1 | | 18.4 | | | 18.2 | | 16.7 | 17.5 | | 0.486 | | 0.863 | | 0.767 | | | 0.894 | | 1.000 | | 0.982 | | 0.925 | | 0.987 | | 0.992 |  |
| Clostridiaceae | 2.3 | | | | 1.9 | | 2.0 | | 2.2 | | 2.0 | | | 2.0 | | 1.8 | 2.6 | | 0.484 | | 0.160 | | 0.176 | | | 0.964 | | 1.000 | | 0.441 | | 0.956 | | 0.210 | | 0.462 |  |
| Clostridiales_unclassified | 0.1 | | | | 0.1 | | 0.1 | | 0.1 | | 0.1 | | | 0.1 | | 0.1 | 0.1 | | 0.652 | | 0.083 | | 0.907 | | | 0.977 | | 0.647 | | 0.787 | | 0.402 | | 0.544 | | 0.995 |  |
| Corynebacteriaceae | 0.0 | | | | 0.0 | | 0.0 | | 0.0 | | 0.0 | | | 0.0 | | 0.0 | 0.0 | | 0.099 | | 0.156 | | 0.358 | | | 0.951 | | 0.348 | | 0.998 | | 0.136 | | 0.984 | | 0.265 |  |
| Dermabacteraceae | 0.0 | | | | 0.0 | | 0.0 | | 0.0 | | 0.0 | | | 0.0 | | 0.0 | 0.0 | | 0.166 | | 0.259 | | 0.500 | | | 0.955 | | 0.575 | | 0.998 | | 0.286 | | 0.988 | | 0.460 |  |
| Desulfovibrionaceae | 1.2 | | | | 1.0 | | 1.0 | | 1.2 | | 0.9 | | | 1.1 | | 1.1 | 1.3 | | 0.122 | | 0.288 | | 0.992 | | | 0.679 | | 0.868 | | 0.254 | | 0.985 | | 0.875 | | 0.688 |  |
| Eggerthellaceae | 0.4 | | | | 0.2 | | 0.4 | | 0.3 | | 0.2 | | | 0.6 | | 0.2 | 0.3 | | 0.002 | | 0.000 | | 0.007 | | | 0.983 | | 0.000 | | 0.801 | | 0.000 | | 0.583 | | 0.000 |  |
| Enterobacteriaceae | 0.6 | | | | 0.4 | | 0.7 | | 0.4 | | 0.5 | | | 0.9 | | 0.4 | 0.4 | | 0.070 | | 0.274 | | 0.226 | | | 0.970 | | 0.362 | | 0.951 | | 0.170 | | 1.000 | | 0.145 |  |
| Enterococcaceae | 0.05 | | | | 0.10 | | 0.12 | | 0.02 | | 0.15 | | | 0.10 | | 0.05 | 0.001 | | 0.024 | | 0.234 | | 0.956 | | | 0.337 | | 0.809 | | 0.072 | | 0.852 | | 0.848 | | 0.379 |  |
| Erysipelotrichaceae | 1.6 | | | | 1.2 | | 1.6 | | 1.3 | | 1.2 | | | 1.9 | | 1.2 | 1.3 | | 0.272 | | 0.154 | | 0.273 | | | 1.000 | | 0.282 | | 0.995 | | 0.281 | | 0.995 | | 0.405 |  |
| Eubacteriaceae | 0.2 | | | | 0.1 | | 0.2 | | 0.2 | | 0.1 | | | 0.3 | | 0.1 | 0.2 | | 0.439 | | 0.116 | | 0.626 | | | 0.997 | | 0.457 | | 0.937 | | 0.343 | | 0.860 | | 0.805 |  |
| Firmicutes_unclassified | 19.7 | | | | 20.6 | | 20.7 | | 19.6 | | 21.2 | | | 20.2 | | 20.0 | 19.3 | | 0.553 | | 0.628 | | 0.907 | | | 0.957 | | 0.973 | | 0.869 | | 1.000 | | 0.994 | | 0.986 |  |
| Lachnospiraceae | 8.5 | | | | 8.5 | | 9.1 | | 7.9 | | 9.7 | | | 8.5 | | 7.4 | 8.4 | | 0.064 | | 0.897 | | 0.089 | | | 0.062 | | 0.554 | | 0.483 | | 0.597 | | 0.668 | | 0.999 |  |
| Lactobacillaceae | 0.9 | | | | 0.5 | | 1.1 | | 0.3 | | 0.6 | | | 1.6 | | 0.4 | 0.2 | | 0.000 | | 0.048 | | 0.004 | | | 0.871 | | 0.004 | | 0.450 | | 0.000 | | 0.884 | | 0.000 |  |
| Lactobacillales_unclassified | 0.0 | | | | 0.0 | | 0.0 | | 0.0 | | 0.0 | | | 0.0 | | 0.0 | 0.0 | | 0.560 | | 0.197 | | 0.560 | | | 0.839 | | 0.541 | | 0.541 | | 0.956 | | 0.956 | | 1.000 |  |
| Microbacteriaceae | 1.0 | | | | 0.5 | | 0.7 | | 0.9 | | 0.4 | | | 1.0 | | 0.7 | 1.1 | | 0.548 | | 0.045 | | 0.562 | | | 0.834 | | 0.255 | | 0.249 | | 0.733 | | 0.725 | | 1.000 |  |
| Morganellaceae | 0.0 | | | | 0.0 | | 0.0 | | 0.0 | | 0.0 | | | 0.0 | | 0.0 | 0.0 | | 0.339 | | 0.339 | | 0.308 | | | 1.000 | | 0.498 | | 1.000 | | 0.526 | | 1.000 | | 0.498 |  |
| Oscillospiraceae | 0.2 | | | | 0.2 | | 0.2 | | 0.3 | | 0.2 | | | 0.2 | | 0.3 | 0.2 | | 0.070 | | 0.411 | | 0.430 | | | 0.252 | | 1.000 | | 0.885 | | 0.242 | | 0.661 | | 0.876 |  |
| Peptococcaceae | 0.0 | | | | 0.0 | | 0.0 | | 0.0 | | 0.0 | | | 0.0 | | 0.0 | 0.0 | | 0.240 | | 0.226 | | 0.043 | | | 0.109 | | 0.103 | | 0.330 | | 1.000 | | 0.929 | | 0.921 |  |
| Prochloraceae | 0.4 | | | | 0.5 | | 0.3 | | 0.5 | | 0.4 | | | 0.2 | | 0.6 | 0.5 | | 0.129 | | 0.393 | | 0.574 | | | 0.899 | | 0.744 | | 0.962 | | 0.333 | | 0.997 | | 0.449 |  |
| Rikenellaceae | 6.0 | | | | 6.9 | | 5.4 | | 7.4 | | 6.3 | | | 4.6 | | 7.4 | 7.5 | | 0.085 | | 0.474 | | 0.437 | | | 0.900 | | 0.712 | | 0.883 | | 0.308 | | 1.000 | | 0.287 |  |
| Ruminococcaceae | 36.9 | | | | 36.1 | | 35.8 | | 37.2 | | 35.7 | | | 36.0 | | 36.6 | 37.8 | | 0.431 | | 0.654 | | 0.778 | | | 0.984 | | 0.999 | | 0.814 | | 0.995 | | 0.954 | | 0.871 |  |
| Streptococcaceae | 0.6 | | | | 0.3 | | 0.7 | | 0.2 | | 0.4 | | | 1.1 | | 0.3 | 0.2 | | 0.096 | | 0.321 | | 0.158 | | | 0.998 | | 0.323 | | 0.960 | | 0.237 | | 0.990 | | 0.134 |  |
| Sutterellaceae | 0.0 | | | | 0.0 | | 0.0 | | 0.0 | | 0.0 | | | 0.0 | | 0.1 | 0.0 | | 0.243 | | 0.243 | | 0.243 | | | 0.349 | | 1.000 | | 1.000 | | 0.349 | | 0.349 | | 1.000 |  |

*Note:* *n* = 12 birds/group/room. *P*-values less than 0.05 are highlighted in green.
